# Precepts of Universal Medical Practice

**Published:** 1844-10

**Authors:** 


### ART. III.

*Praxeos Medicæ Universæ Præcepta.* Auctore JOSEPHO FRANK, Joannis Petri Filio, &c.

*Precepts of Universal Medical Practice.* By JOSEPH FRANK, Son of John Peter Frank.

*Partis Tertiæ Volumen Secundum, Sectio Prima, continens Doctrinam de Morbis Tubi Intestinalis.* Quam exposuit FREDERICUS AUGUSTUS BENJAMIN PUCHELT, Magno Duci Badarum à Consiliis Aulæ intimis, &c.—*Leipsiæ*, 1841.

*The Second Volume of the Third Part, the First Section, containing the Treatise on Diseases of the Intestinal Canal.* By FREDERIC AUGUSTUS BENJAMIN PUCHELT, Privy Counsellor to the Grand Duke of Baden, &c.—*Leipsic*, 1841, pp. 786.

THIS work is not what the first part of its title declares it to be, the production of the celebrated Joseph Frank ; but that of Professor Puchelt of Heidelberg. The latter in a brief preface informs us that Frank in 1827, proposed to him to undertake that portion of the great work on practical medicine which should comprise the diseases of the intestinal canal ; and the volume before us, containing nearly 800 pages, is the result of his compliance with the proposal; advice and certain notes communicated by Frank being the amount of the learned editor's obligations in that quarter. It will be our place to examine how far he has shown himself worthy of the delegated trust.

The following is his arrangement of the very extensive subject intrusted to him : Congenital malformation of the intestines, and their abnormal form and situation; enteritis, suppuration ulceration, tenuity (marcor,) and perforation of the bowels; induration of their membranes, and tubercles, melanosis, medullary fungus, scirrhus and cancer in their tract; morbid adhesions, polypi, œdema, hydatids, and intestinal stricture and contraction ; worms; flatulent affections ; colic ; constipation ; ileus ; diarrhea ; dysentery ; hemorrhages ; and cholera. Each section of the work, and several of the subsections are prefaced by a literature of the matter in hand as copious as possible, ranging from the remotest antiquity to the last publication of Louis, or a recent number of our own Review. The author's addiction to literature, which is certainly intense, does not, however, divest him of sound practical judgment. From the days of Sydenham, a notion, founded apparently on a jocular remark of that illustrious man to Sir Richard Blackmore, has pervaded a portion of our profession that these qualities are incompatible. But the following passage shows that indulgence in research has not impaired the learned Professor's just and practical appreciation of his subject :

"It has occasionally happened that intestinal diseases have been considered matters of little moment, by the Brunonians for instance ; but it more frequently occurs that physicians seek in the abdomen a disease which exists elsewhere, and that the intestines are considered the cause and seat of almost all maladies ; now they accuse crudities, now repletion, and now gastro-enteritis. Be such exaggeration far from us ! Let us not attach too much importance to individual symptoms ; to those derived from the tongue for example, the redness of which has been accounted a certain sign of gastro-enteritis, and which when coated has been thought to indicate crudities. Let us not confound together those sympathetic, secondary and symptomatic affections which occur in almost every disease, with those which are idiopathic, primary and essential. On the other hand let us beware lest intestinal diseases should be overlooked, and estimated at less than their real value! Therefore it is the duty of the physician, free from preconceived opinion, and the empire of hypothesis, to consider with an unbiassed mind all the symptoms, their succession and composition, not neglecting due examination of the abdomen, as well externally by compression and percussion, as internally by the introduction of the finger, a probe, or speculum within the anus; and to weigh the causes of the disease, the epidemic and individual constitution, the family, age, habits, temperament, and preceding diseases of the patient, and the medicines already administered to him." (pp. 15, 16.)

ENTERITIS. The subject of enteritis receives ample treatment at the Professor's hands. Nowhere have we found it more learnedly discussed, or with more practical good sense. His divisions of the disease are numerous, for besides the great distinctions into serous and mucous enteritis we have the inflammation considered with regard to the portion of intestine implicated. Where it occupies the greater part of the tract of the intestines, it constitutes the *E. diffusa*, which may at its commencement be either the serous or mucous variety; in its progress it may affect all the tunics. This form of enteritis is scarcely ever due to a local cause, excepting this be poison; but arises, for the most part, from an universal diathesis and metastasis. *E. circumscripta* may arise in any portion of intestine from a local mechanical cause, such as hernia or intus-susception. Inflammation of the duodenum forms a division, and inflammation of the small intestines generally, equivalent to the *enteritis iliaca* of Sauvages, the *febris iliaca inflammatoria* of Hoffman, and the *jejuno-iliotis*, or *ileo-jejunitis* of Broussais, constitutes another. We have the inflammation of the *cecum*, designated by our author *typhlitis*; that of the vermiform appendix, and subsequently that peculiar affection adverted to by P. Frank, but first described as a distinct disease by Dupuytren, in which the cellular membrane situated between the *cecum*, and the *iliacus internus* muscle is the seat of inflammation, which in its progress is wont to extend to the intestine, and terminate in suppuration. This Puchelt calls *peri-typhlitis*. The affections of the other large intestines are described under the names of *colitis*, and *architis* (inflammation of the *rectum*), and that inflammation of the cellular membrane surrounding the gut whence fistula arises, is described, but not, as it perhaps should have been consistently with his own nomenclature, under the designation of *peri-architis*. Of his *enteritis serosa*, he remarks that it has not its sole seat in the serous covering of the intestines, but that the abdominal peritoneum is involved, and that the disease is properly referred to peritonitis. There is some confusion of idea, and consequently of nomenclature pervading the profession on this head. When the disease occupies chiefly, as it does in puerperal females, the abdominal peritoneum, the bowels being amenable to medicine, we should call it peritonitis. When, however, the intestinal peritoneum is at first the seat of the inflammation, the muscular tunic, as it generally does, partaking of the affection, the bowels being obstructed, we should apply to it one of the names suggested by Abercrombie, viz., *E. peritoneo-muscularis*, or *seroso-muscularis*. We are aware that we have high continental authority, (that of Louis for instance, who considers the only real enteritis to be that associated with diarrhea,) against us. But we regard the ancient nomenclature, modified in the one form of enteritis by the affix we have selected from Abercrombie, or for brevity's sake, by the word *serosa*, and in that of the other by the term *mucosa*, at once more in accordance with the characters of the respective diseases, and with anatomy, (for we have been taught to consider their peritoneal coat as a portion of the intestines,) than the modern innovation.

After the discussion on E. serosa, we have E. mucosa, considered under two heads, viz., as it affects grown persons, and infants, the latter constituting the *E. mucosa infantum* of Abercrombie. *E. totalis* is then described, not as an affection, as the name might seem to imply, of the whole tract of the intestines, but of the entire tunics of any given portions. We then have the general division of the various forms of *enteritis* into acute and chronic, and next follow in order the following species: E. traumatica, phlegmonosa, erysipelacea, and exanthematica, rheumatica, arthritica, hemorrhoidalis, catarrhalis, scrofulosa, carcinomatica, nervosa, and putrida, which last, the author says, is conjoined with petechiæ and other putrid symptoms, and is prone to terminate in gangrene.

We are thus presented with twenty-eight varieties or species (as we may choose to view them) of this one disease. Here is certainly division enough and to spare; but in truth, in judging of German medical or other literature, allowance must be made for the peculiarities of the German mind, its tendency at one time to discern or almost create distinction where nature has established little or none; and at another to discover relations imperceptible to other eyes, and to associate into one vast system the "discordia semina rerum." These peculiarities, however, merit forgiveness on the score of the singular industry, learning, profundity and other high qualities of this truly gifted people.

As specimens of the author's manner we select his descriptions of two forms or varieties of enteritis, viz., typhlitis and perityphlitis:

"Aretæus, Celsus, Bonetus and Fabricius Hildanus have observed the *intestinum cæcum* to be primarily affected with the inflammation which we have called *typhlitis*. Van Swieten, and J. P. Frank, have likewise noticed this disease; but Unger, Copland, Posthuma, Holscher, Albers, and Burne have described it more accurately. Foreign bodies, such as plum- or cherry-stones, hardened feces, or worms, excite this inflammation. Morgagni has seen the cecum after a fall, distended with excrements and inflamed, and Speir has observed it ruptured and inflamed in consequence of external violence; and there can be little doubt, that chilling of the body occasionally gives rise to the disease. Finally, inflammation of the cellular coat situated behind the *cecum*, and of the vermiform appendix may, either of them, extend to the *cecum*. Some obstruction of the bowels having preceded, a pain more or less severe arises in the right iliac region, which is increased by pressure and motion of the body, so that the patient desires to keep it in a state of repose; sympathetic pains extend, on any movement of the body, from the sacral region to the right thigh, and the testicle is drawn up to the abdominal ring. There is fever which in stercoral typhlitis is moderate, but in other forms of the disease is very severe; vomiting sometimes supervenes, so too do symptoms of ileus. The pains are less severe, when the mucous membrane alone is inflamed, nor are the bowels obstructed, but on the other hand a mucous and bloody diarrhea prevails, with gripes and colic pains preceding each evacuation. Typhlitis passes not unfrequently to the cellular tunic behind the cecum, sometimes to the peritoneum, or to other portions of the intestinal tube; likewise it may continue in a chronic form, and leave behind it induration of the intestine." (pp. 147-50.)

Passing over his description of inflammation of the vermiform appendix, we proceed to transcribe that of perityphlitis, omitting only, for brevity's sake, Dr. Puchelt's abundant marginal references to the publications, (be they original works or periodicals,) and the pages of the publications, in which the observations of the authorities he quotes have been published.

"Very frequently the cellular membrane, situated between the blind intestine and the iliac muscle is seized with inflammation, which often passes to the intestine itself, and terminates in suppuration. This disease we regard as peculiar, and have called it perityphlitis. P. Frank alluded to it, but Dupuytren first regarded it as a distinct disease. After his day, Husson and Dance, Meniere, Abercrombie, Unger, Pezerat, Téalier, Goldbeck, Corbin, Ferrall, Puchelt, Copland, Truchsess, Smith, Burne, Posthuma, Wilhelmi, Albers, Piotag, Lebotard, Bricheteau, Grisolle and others, have frequently observed this disease, and fully described it. It arises most frequently from cold; and besides may be excited by a damp dwelling house, sordes of the first passages, worms, spirituous liquors, preparations of copper and lead, disturbance of the uterine system, child-bearing, emetics and purgatives, riding on horseback, and affections of the mind. After a feeling of indisposition of a few days' duration, and loss of appetite, white tongue, eructations, nausea, and obstructed bowels or diarrhea, there arises excruciating, pungent and wandering pain of the abdomen, felt in the epigastric region and other points; the abdomen being painful on pressure but soft, the bowels costive. Before four and twenty hours have elapsed, the pain passes to the right iliac region, and becomes fixed there. Sometimes it extends to the urinary bladder, in which case, the urine, ordinarily pale, becomes thick, of a reddish brown colour, sparing in quantity, and heat is felt in discharging it. By pressure not only is the circumscribed pain in the iliac region increased, but a tumour is perceptible, elastic at first, afterwards possessing a considerable degree of hardness. Motion of the body, and especially that of the right lower limb, increase and aggravate the pain so much, that patients generally lie on the back or right side, without changing their position, with the feet drawn up. There is fever, a dry and hot skin, paleness of the face, bowels rarely in a liquid state, generally costive; sometimes vomiting is present, but rarely *ileus*;* nevertheless, expulsion of flatus takes place, some increase of the cecal pain preceding such expulsion. Patients remain in this state for some days, till the disease terminates either by treatment, by crisis or suppuration. When crisis takes place by urine and perspiration, there remains frequently a certain degree of sensibility in the affected part, an intermitting pain, a sense of fulness, and likewise a degree of positive intumescence for some time. When an abscess takes place in the cellular substance behind the *cecum*, the pus may be effused into the *cecum* itself, healthy purulent matter being excreted *per anum*, and the patients being speedily restored to health. The matter sometimes but very rarely finds issue through the urinary bladder, uterus or vagina. There is sometimes fluctuation in the anterior surface of the iliac region, or in the lumbar region behind the crest of the *os ilium*, whence arise ulcers and fistulæ, producing hectic, communicating at once externally, and in the cecum, especially fatal, often so by means of stercoraceous effusion into the peritoneum, and peritonitis. In such cases the inflammation has extended from the

* The author uses this term in the sense of stercoraceous vomiting, or (to take his own definition) it is "a disease in which feces are scarcely or not at all expelled *per anum*, but are vomited, pain and anxiety being accompanying symptoms." We think that our very learned author would have done better had he given the precise symptoms of every disease he treats of, rather than comprehended any portion of them under a term which we consider as considerably vague. He treats of ileus or iliac passion as a distinct disease. It is not this; but an assemblage of symptoms, varying in different cases, indicative of more than one disease of the intestinal canal; violent spasm, permanent stricture, intus-susception, enteritis, &c.—REV.

cellular membrane to the cecum, vermiform appendix and peritoneum, and these parts have been found inflamed and disorganized. When this occurs, it may be a matter of question where the inflammation has been primarily seated. Wilhelm is of opinion that the inflamed cellular tissue may become gangrenous, though no case is given which proves it. An observation, however, of his own seems to prove it.* I was present, and saw the dissection; the cellular tissue was rather destroyed by sphacelus than distended with pus. Certainly no abscess had been formed. Finally, we must remark that cachectic subjects attacked with this disease scarcely ever escape suppuration; but in florid and robust subjects by proper treatment it may be easily and safely prevented. In the former class the disease passes into the chronic state, in the latter its course is sometimes very brief." (pp. 152-7.)

Two cases are referred to by M. Puchelt, in which perfect recovery took place from the very deplorable circumstance of stercoral fistula from perityphlitis. The one is quoted from Vogel, (Handb. iv bd. p. 321,) the other from Jabgerschmidt, (Ephem. naturæ curios. dec. iii, b. ii, obs. 156, p. 245.)

When he comes to the subject of treatment, we find our author's practical precepts judicious. He advises that the force of the disease should be broken by antiphlogistic measures proportioned to its severity. He would not allow the small, weak, and contracted pulse so common in this disease to be an obstacle to bloodletting, or to its repetition in diffuse, serous, total, acute, traumatic, phlegmonous, or hemorrhoidal enteritis. This decision is the more creditable, since his countrymen Hildenbrand, quoted by him, deems lightly of the influence of venesection in enteritis, and has not much faith in leeches. Supported, however, by authorities extending from Hippocrates and Aretæus through Quarinus, Fordyce, Hoffman and Bang, to Abercrombie, the learned Professor adheres to (what we deem) the orthodox practice, but subjoining, "ne quid nimis!" We have a crisis to come, and blood must be spared that this may be accomplished. We think the word crisis vague, and we know that amongst continental physicians it is made a plea for feeble proceedings. It is true that we cannot at once cure any inflammation; that peculiar condition of the capillaries which is the very essence of this affection, cannot be annulled at a blow. What we really effect by bloodletting and other antiphlogistic measures is the placing of the inflammation within the sanatory power of the constitution. By such measures, the dilated and distended vessels commence the process of contracting and unloading themselves, and the resorption of effused fluids is begun. The completion of the process may require days, and during this period additional means of the same nature as those originally employed may be demanded to accelerate it if tardy, or to combat some augmentation of the morbid condition. How often have we watched the course of this process in the eye! Now what is called crisis, the increased secretion of urine, the relaxation of the cutaneous exhalents, the general relaxation indeed of the system, does not appear to us to be the means by which nature accomplishes her work, but an indication that she has accomplished it, or nearly so.

With regard to internal measures, we are cautioned against saline or other matters which may irritate by contact, but are advised to give oily

* Diss. de Perityphlitide, Heidelberg, 1837, p. 16.

and demulcent matters, such as almond emulsion, oil of almonds recently prepared, and decoctions of pearl-barley, oatmeal, and marshmallow. Fomentations of infusion of chamomile flowers, or of milk with soap, and oily frictions to the abdomen are recommended. Semicupia and the tepid bath are advised in perityphlitis and other cases of partial enteritis; but where there is extreme anxiety and restlessness they are thought (justly) to increase distress. Dr. Puchelt would limit the use of cold fomentations recommended by Smith, Abercrombie and H. Wolff, and the internal use of ice suggested by Arnster, to traumatic and phlegmonous enteritis. The employment of emollient and oily enemata is suggested. though we are told that even these should be avoided when the lower part of the intestine is inflamed. Metastatic derivatives, such as sinapisms and blisters to the inferior extremities, and the surface of the abdomen are recommended at the commencement of the disease, and the author intimates that metastatic derivation is the principle on which bloodletting and enemata are beneficial. Without entering into a lengthened argument on the matter, we take the liberty of remarking that the latter point is very questionable indeed. When the force of the inflammation is broken, and loss of blood is of no further avail, calomel should be resorted to. It is of the greatest use in serous enteritis, but of less in the mucous and total variety. Where it is counter-indicated, he suggests the employment of mercurial frictions to the abdomen. Severe pain should be allayed by opium, hyoscyamus, or aqua laurocerasi, especially one of the two latter, they being exempt from the constipating effect of opium. We are counselled to employ laxatives of the milder kind in the advanced stage of the disease. Those recommended are castor-oil, sulphate of magnesia in emulsion or gum-water; and where bile abounds, cream of tartar and pulp of tamarinds. Such are the general therapeutic precepts of Professor Puchelt for the treatment of this very dangerous disease. Certain modifications are suggested adapted to its different varieties, such as common sense would suggest to most practitioners.

These practical precepts for the management of enteritis, excepting some difference in form, correspond very precisely with the means resorted to in this country. We coincide in his cautions regarding certain points of practice, especially in that regarding the warm bath. We do not know a more barbarous proceeding than the taking of a patient suffering under some acute and it may be extensive phlogosis from his bed to plunge him into a bath. The unavoidable motion and exertion aggravate pain, and accelerate the circulation, whilst the latter effect is produced likewise by the heat of the bath. We have never known such a proceeding otherwise than injurious under the circumstances described; and in more than one case it has appeared to contribute very much to the fatal result. In acute articular rheumatism, to which disease there seems an idea to prevail that it is especially applicable, we have found it especially pernicious. Independent of the disturbance and exertion very injurious in a class of diseases of which repose is one of the most important remedies, the patient is at once transferred from a temperature of from 65° to 70°, to one of 98°, and the latter is applied through the denser medium which transmits it the more rapidly to the body. What but an exciting effect, under circumstances demanding abatement of excitement, can be expected; and such we have ever found to follow.

The author's language regarding the use of calomel in the serous form of enteritis will coincide with the views of British physicians, and is such as certainly a few years ago we should not have heard from any point of the continent.

DYSENTERY. The character of the chapter on dysentery leads us to bestow on it especial notice. It is certainly the most learned and elaborate treatise on the disease we have ever seen. It extends over 103 pages, twenty-six of which are occupied by a literature of the disease, commencing with Hippocrates, and terminating in the third volume of the Transactions of the Medical and Physical Society of Calcutta, published in 1835, and comprising references to the independent works and journal communications of above 1000 writers. It is quite supposable that a considerable per centage of the works referred to may have not contributed much to the abatement of the ravages of the disease; but that such a phalanx of writers should have chosen this theme is evidence of the magnitude that this scourge of armies has possessed in the eyes of the profession in all ages of the world. After a very copious and accurate description of symptoms we are presented with the necroscopy as furnished by Rokitanski. This writer classes the morbid appearances under four heads, according to their intensity. In the first class we learn that under a thin ash-red coloured mucus, the mucous lining of the great intestine is shown swollen and red in lines (striæ); and the epithelium displays either vesicles or furfuraceous scales, under which the mucous coat appears broken, and may be removed in the form of a pulp. The exudation from the intestine is acid. In the second form the same process has extended over a greater space, the mucous membrane in many places being covered with an ash-coloured, tenacious mucous, and itself being transformed into a gelatinous substance which may easily be detached. At the same time many protuberances, arising from serum effused into the submucous coat are visible; the affected part of the intestine is dilated, distended with gases and brown-coloured fluid, its membranes are thickened, and the follicles are marked either by inflammation, copious secretion or softening. In the third variety, these protuberances (arising from effusion into the submucous tissue) are so accumulated, that the internal surface (of the intestine) appears unequal, and studded with eminences (clivosa); the mucous membrane covering these tubercles is partly in the same condition as in the second form, partly changed into a crust firmly adhering, obscurely red, stained with blood, or of a greenish hue, or again, it is entirely destroyed so that the submucous coat appears almost naked. The tubercles are sometimes confluent. The intestine contains a brown, ichorous, fetid, and flocculent matter. In the fourth and last condition, when the disease has attained the highest degree, ample tracts of mucous membrane are changed into a black, friable and, as it were, burnt mass; the submucous coat is seen in the first place full of dark blood, and a *seroso-bloody* fluid, or pale and containing in its vessels a black, pulverulent blood, or finally it is full of pure pus. The affected part of intestine contains within it a cadaverous, brownish black fluid like coffee, and is either dilated as in the former cases, or collapsed, the muscular coat being denser than natural, pale, ash-coloured, elastic and very friable. The serous membrane has lost its brilliancy, it is full of injected vessels, and is covered with a brown ichorous exudation; the mesocolon and the mesentery itself are occasionally in a similar condition; the mesocolic glands are swollen, obscurely red, and turgid with blood. Not unfrequently the morbid process extends beyond the valve of the colon, so that the mucous membrane of the small intestine is affected in a slighter degree than the colon.

Besides this ample detail, Professor Puchelt deems it necessary to add his own observations, closely corresponding with those of Rokitanski, and quotations from various writers, especially our own, on a portion of the subject not sufficiently described by this writer, but well known to army medical men as the source of that distressing and fatal disease, chronic dysentery, intestinal ulcers. His references on this branch of the subject are to Christison, (Edin. Med. and Surg. Journal, vol. xxxi, p. 217,) to Smith, (Ibid. xlii, v. p. 345,) and to Cheyne, (Dublin Hospital Reports, vol. iii, pp. 28, 32.) The two latter, Smith and Cheyne, mention perforation of the intestine as an occasional result of such ulcers, though Smith had seen but one example, in the transverse arch of the colon, and this to so small an extent, that no effusion into the peritoneum had occurred. Cheyne mentions its having sometimes occurred unexpectedly, and its having been fatal from effusion. Gintrac (Journal de Méd. Pr. de Bordeaux, Jan., 1839,) depicts a singular species of dysentery, in which the mucous crypts of the large intestine were affected in the same way as the segregated and agminated glands of Peyer are in typhus.

We should have considered the evidence from these morbid appearances, connected with the well-known symptoms of the disease, as conclusive of its inflammatory nature, and that the inflammation is primarily and essentially seated in the mucous lining and glandular follicles in the colon and rectum. This view, which all our experience of the disease shows to be the only correct one, is here included among various other opinions of the nature of the disease, and is supported by the authorities of Fournier and Vaidy, Broussais, Foderé, Neuman, Müller, Jaeger, and Jarwandt. The author's opinion approaches to this, so far as we can discern it through the not very clear medium of the language in which it is conveyed. The following is his own account of it:

> "I should think that the blood, in the first place contaminated, or in some degree poisoned by a *miasm*, produces in the intestine, especially in the colon and rectum, a peculiar condition which may be compared to an eruption (exanthemat), and which may pass into inflammation, ulceration, induration and gangrene, and produce either simple, inflammatory, nervous, or putrid fever. But I willingly confess that this opinion, equally with others, reposes less on observation than on inference." (Note, p. 644.)

Besides inflammation, or such an approach to it through the two successive stages of contaminated blood, and an exanthematous condition, as our Professor selects, we have dysentery considered, and on able authority, as rheumatism, catarrh of the intestines, spasm of the great intestines (Cullen,) erysipelas, acrid bile, fever turned inwards on the intestines, (Sydenham,) and as a primary affection of the nervous system, the ganglionic system, and of the veins. Quitting, however, this branch of the subject, and passing over his very learned disquisition on the varieties of the disease, of which he enumerates thirteen, quoting very largely from British writers, particularly on the subject of tropical dysentery, we hasten to examine the section which comprises his method of cure.

On this branch of the subject we have much learning, very much reference to the cerated glass of antimony, and to the purchase by Louis XIV of the secret remedy of Helvetius (which proved to be ipecacuanha), and other matters of much moment, in all probability, in a day gone by; but we do not find a rational plan of proceeding suggested, such as in the present state of medical science might have been expected. The vagueness of the Professor's own opinion of the nature of the disease diffuses itself through the section on treatment. He tells us that he would treat dysentery as he would smallpox, scarlatina, continued fever, or typhus; that any primary plan of treatment, which may break the force of the disease, not being clearly indicated, we must acquiesce in a mode of proceeding adapted to the symptoms, unless we choose to act on this or that hypothesis or preconceived opinion. We think that not only has pathological research, but—what we deem more deserving of reliance—clinical observation been employed in vain, unless we have attained to some principles in the treatment of this disease. It is true that the actual practice may vary in force and, in some degree, in kind in individual cases; yet some principles, some adaptation to a recognized pathological condition should be discernible in it.

Of general bleeding he speaks with excessive reserve, questioning whether it abates the intensity of dysentery as it does that of other inflammations, and expressing the opinion, that in a mild disease it is superfluous, and in a debilitated and exhausted frame and a malignant dysentery it is injurious. The whole weight of his own doubts is thrown into the scale against bleeding; yet in its favour he quotes the highest names in medicine—Sydenham (himself a host), Huxham, Akenside, Zimmermann, Mursinna, Rademacher, Somers, Bampfield, Annesley, Antenrieth, Mautz, Hauff, Cheyne, Juncker, and P. Frank. This last writer states that he had on one occasion bled a patient in dysentery three times with a successful result; and the same distinguished authority mentions the prevalence at Strasbourg of an epidemic of dysentery which was exceedingly fatal until medical men resorted to bloodletting. Our own experience is in accordance with the views of these distinguished parties. Many cases, certainly, have we seen successfully treated without bloodletting; but in severe cases abroad and in the provinces of this country, where general excitement of the system has been associated with indications of severe local affection we have found general bloodletting very beneficial, and in some such cases, as it appeared to us, essential to the cure. Leeches he approves of, once or even more frequently applied, provided architis (inflammation of the rectum) be existing, or peritonitis impending; and recommends the anus, perineum, or sacral region as suitable points for their application. Of opium he speaks with general praise as a remedy of dysentery, saying that there scarcely exists a case in the treatment of which it may not find place. He quotes the examples of British writers—Christison, Smith, and Cheyne—who have themselves administered and recommended to others very large doses of this drug in this disease. But he deems it necessary at the close of his quotation to insert a caution, implying censure on the practice of these writers. "Beware," says he, "of following these examples without due consideration, for they show rather what the human frame can bear than what ought to be done."

On the subject of calomel as a remedy of dysentery, he confines his information to what has been delivered by British writers; and these, with his characteristic industry and research, he quotes in abundance, from the days of Cleghorn to those of the Johnsons, Annesleys, and Ballinghalls of the present time. But no comment of his own does he offer, either on what has been called " the scruple-dose practice of India," or the more moderate proceedings adopted in temperate climates. For our own parts we acknowledge the value of mercury, associated with depletion—general, local or, both—in dysentery, as we do in other inflammatory diseases; but we prefer in this disease the forms less calculated to irritate the bowels, such as the gray oxyde, conjoined with opium and ipecacuanha, as they exist in Dover's powder.

Next follows an enormous list of tonics, mucilaginous and bitter, astringent and bitter, merely bitter, and those called antidysenteric. Of these medicines, vegetable and mineral, *we* find a difficulty in referring a considerable proportion to any recognized class in the *materia medica.* The author acknowledges that many of them have been introduced lest he should appear to have omitted anything; a charge very unlikely indeed to lie against our learned author.

We now take leave of Professor Puchelt, without, however, professing to have given a complete review of his work, but only specimens of his manner of discussing the more important and familiar of the diseases belonging to the department of general pathology allotted to him. To whom would we recommend this, the completest treatise on diseases of the alimentary canal we ever saw? Not to the practitioners of busy England: something more compendious is required to answer their prompt demands. But we can recommend it to the medical *literateurs* of this or any other country, as a wholesale storehouse, whence materials may be drawn for the purposes of retail doctors, both in town and country. Might it not merit the notice of the Sydenham Society? By one feature of the work our love of country (no discreditable feeling, we trust) has been much gratified—the very large proportion of its vast wealth that is derived from British sources.

---

